# Targeting Her2/neu in uterine serous carcinoma: A paradigm shift in management

**DOI:** 10.18632/oncotarget.26413

**Published:** 2018-11-30

**Authors:** Salvatore Lopez, Burak Zeybek, Alessandro D. Santin

**Affiliations:** Department of Obstetrics, Gynecology, and Reproductive Sciences, Yale University School of Medicine, New Haven, CT, USA

**Keywords:** endometrial cancer, uterine serous carcinoma, USC, HER2/neu, trastuzumab

Endometrial cancer (EC) is the most common gynecologic malignancy in the United States; approximately 63,230 newly diagnosed cases and 11,350 deaths from the disease are expected in 2018 [1]. It is typically categorized as Type I and Type II; type I tumors have more favorable outcomes due to endometrioid histology, early stage at diagnosis, retention of hormone receptor status and younger age at onset. On the other hand, Type II tumors portend a poorer prognosis, as these tumors are histologically high-grade (serous or clear cell) with no hormone receptors and they mostly present at an advanced stage.

Uterine serous carcinoma (USC) is a rare (3%-10% of all endometrial cancers) but highly aggressive histologic type with an overall survival of 18% to 27% [2]. Traditionally, the mainstay of treatment has been a combination of comprehensive surgical staging, followed by platinum plus taxane combination chemotherapy. However, response rates to this traditional approach, especially in advanced and recurrent disease, have been lower than expected and short in duration, revealing the necessity for novel therapies. In order to find new and effective therapeutic targets, the genetic landscape of USC has recently been comprehensively investigated [3, 4]. Whole exome sequencing (WES) and confirmatory immunohistochemistry (IHC) studies have revealed that up to 35% of USC patients harbor c-erbB2 gene amplification and/or human epidermal growth factor receptor 2 overexpression (ie, HER2/neu, the receptor encoded by the c-erbB2 gene) [4, 5]. HER2/neu provides critical signaling for cell growth, survival, and proliferation, which might at least partially contribute to the poor outcomes [2, 6].

The use of a humanized monoclonal antibody targeting HER2/neu (trastuzumab) as part of a first line therapy in the treatment of HER2-amplified breast cancer has led to the development of clinical trials to evaluate the effectiveness of this agent in endometrial tumors with HER2/neu overexpression. In a cohort of heavily pretreated endometrial cancer patients, Fleming et al., for the first time, evaluated the efficacy of single-agent trastuzumab (Gynecologic Oncology Group, GOG-181B) [7]. Although the results failed to demonstrate any efficacy, this study was criticized due to the fact that HER2/neu amplification was only present in 45% of the patients and the majority of patients harbored Type I tumors (ie, endometrial endometrioid carcinoma) [8].

Several unexpected mechanisms of resistance to trastuzumab have recently been discovered in USC, revealing how the biology of HER2/neu differs in this aggressive tumor from other cancers. For example, in contrast to breast cancer, where HER2/neu is homogenously overexpressed on tumor cells, up to 53% of USC overexpressing HER2/neu at 3+ levels demonstrated high heterogeneity in HER2/neu protein expression by IHC [5]. Furthermore, a high prevalence of PIK3CA hotspot mutations conferring high resistance to single-agent trastuzumab were commonly identified in erbB2 amplified USC [9]. Finally, in multiple reports, shedding of the extracellular domain (ECD) of the Her2/neu receptor and upregulation of CD46, CD55 and CD59 membrane-bound-complement-regulatory-proteins (mCRPs) resulting in resistance to trastuzumab-mediated antibody-dependent-cell-death (ADCC) and complement- dependent-cell-death (CDCD), respectively, were demonstrated [9]. These findings not only provided potential molecular explanations to the failure of previous NRG/NCI cooperative clinical trials targeting endometrial cancer with single-agent trastuzumab, but also supported the hypothesis that a combination of trastuzumab with chemotherapy might represent the most effective therapeutic strategy for tumors with heterogenous overexpression of HER2/neu such as USC.

Fader et al. recently conducted a multicenter randomized phase II trial that investigated the effects of trastuzumab in advanced/recurrent USC [10]. In this study, eligible patients, who had primary stage III/IV or recurrent HER2/neu-positive disease, were randomly assigned to receive carboplatin-paclitaxel (control arm) for six cycles with or without intravenous trastuzumab (experimental arm) until progression or unacceptable toxicity. Among all evaluable patients (*n* = 58), median progression-free survival (PFS) was 4.6 months longer in the experimental group when compared to the control group (8.0 *vs* 12.6 months, *p* = .005; hazard ratio [HR], 0.44; 90% CI, 0.26 to 0.76). Patients with stage III/IV disease (*n* = 41) had the greatest benefit with an 8.6-month-increase in PFS (9.3 months in the control group *versus* 17.9 months in the experimental group, *p* = .013; HR, 0.40; 90% CI, 0.20-0.80). In patients with recurrent disease (*n* = 17), addition of trastuzumab to standard chemotherapy also significantly increased PFS by 3.2 months (6.0 months in the control group *versus* 9.2 months in the experimental group, p = .003; HR, 0.14; 90% CI, 0.04-0.53). Toxicity was not different between treatment arms. Importantly, while overall survival data in the report were not yet mature, a preliminary overall survival analysis of the 36 patients with Stage IIIC/IV disease yielded a 1-sided p value of 0.02 [10].

The results of the study by Fader et al. cannot be overemphasized as they recently led the National Comprehensive Cancer Network (NCCN) to update its guidelines (ie, the addition of trastuzumab to carboplatin/paclitaxel is now the preferred regimen for the treatment of advanced/recurrent USC patients overexpressing HER2/neu); (category 2A recommendation)

(http://www.jnccn.org).

USC are aggressive neoplasms with portend a poor prognosis in a significant number of patients diagnosed with early stage (ie, IA-IB) disease [2, 6]. The positive results of this randomized Phase II study warrant further clinical investigation to determine the efficacy of this novel and well tolerated treatment combination in early stage USC patients overexpressing HER2/neu.
